# Sex differences in management and outcomes of people with ST‐elevation myocardial infarction, New South Wales, 2011–2020: a retrospective cohort study

**DOI:** 10.5694/mja2.70048

**Published:** 2025-09-18

**Authors:** Samia Kazi, Simone Marschner, Haeri Min, Desi Quintans, James JH Chong, Ehsan Khan, David B Brieger, Clara K Chow

**Affiliations:** ^1^ Westmead Applied Research Centre University of Sydney Sydney NSW; ^2^ Westmead Hospital Sydney NSW; ^3^ The University of Sydney Sydney NSW; ^4^ Flinders Medical Centre Adelaide SA; ^5^ Concord Repatriation General Hospital Sydney NSW

**Keywords:** Cardiovascular system, Acute coronary syndrome, Cardiology, interventional, Coronary artery disease, Myocardial infarction

## Abstract

**Objectives:**

To examine whether sex differences in cardiovascular disease care and outcomes for people hospitalised with ST‐elevation myocardial infarction (STEMI) in New South Wales have declined during 2011–2020.

**Study design:**

Retrospective cohort study; analysis of linked New South Wales Admitted Patient Data Collection and Registry of Births, Deaths and Marriages data.

**Setting, participants:**

Adults (18 years or older) admitted to public or private hospitals in New South Wales with STEMI for the first time during 1 January 2011 – 31 December 2020.

**Major outcome measures:**

Proportions of people who received coronary angiography or percutaneous coronary intervention (PCI) within seven days of first STEMI admission, by year and sex; proportions of STEMI admissions followed by major adverse cardiovascular events (MACE) or death (any cause) within twelve months, by year and sex; rate of change in these parameters, adjusted for age group, intensive care unit admission, and Charlson Comorbidity Index score.

**Results:**

We identified 29 435 initial STEMI hospital admissions during 2011–2020; the mean age at admission was 66.1 years (standard deviation, 14.2 years), 8475 patients were women (28.8%) and 20 960 were men (71.2%). The proportions of female patients who underwent angiography (71.9% *v* 85.1%) or PCI (54.4% *v* 70.0%) were smaller than those of male patients. During 2011–2020, the increase in the angiography proportion was greater for female than for male patients (2.7 [95% confidence interval {CI}, 2.5–2.9] *v* 1.5 [95% CI, 1.4–1.6] percentage points per year), as was the change in PCI proportion (3.2 [95% CI, 2.9–3.6] *v* 2.5 [95% CI, 2.3–2.7] percentage points per year). The proportions of admissions followed by MACE (18.4% *v* 15.0%) or death of any cause (14.7% *v* 8.5%) were larger for female than male patients. The decline in the MACE proportion during 2011–2020 was similar for female and male patients (0.8 [95% CI, 0.5–1.1] *v* 0.5 [95% CI, 0.3–0.7] percentage points per year); the decline in all‐cause mortality was greater for female than male patients (1.0 [95% CI, 0.8–1.1] *v* 0.6 [95% CI, 0.5–0.7] percentage points per year).

**Conclusion:**

The increase in the proportions of patients with STEMI who underwent timely angiography and PCI was more rapid during 2011–2020 for female than male patients, and the decline in all‐cause mortality was also greater. Sex differences in treatment and outcomes for people with STEMI are declining but will not be eliminated during the next ten years.

**The known:** The diagnosis, treatment, and secondary prevention of ST‐elevation myocardial infarction (STEMI) heart disease are poorer for women than men.

**The new:** Rates of timely invasive interventions increased more rapidly for women than men with STEMI in New South Wales during 2011–2020, and 12‐month mortality declined more rapidly. However, considerable sex differences in management and outcomes for people with STEMI remain.

**The implications:** Strategies are available for reducing differences in the management of women and men with STEMI, but they will not be completely overcome in the next ten years without more rapid changes.

Cardiovascular disease (CVD) is one of the leading causes of death for both men and women,^1^ but the diagnosis, treatment, and secondary prevention of heart disease is poorer for women.^1^, ^2^, ^3^ The problem is compounded by the perception that CVD is less important for female than male patients.^2^ This problem not only applies to heterogenous conditions such as non‐ST‐elevation myocardial infarction, but also to ST‐elevation myocardial infarction (STEMI), for which a standardised diagnosis and management plan is available.^4^

Sex differences in the treatment of people with acute coronary syndrome and their outcomes have been reported around the world. A study in twelve European countries found that 30‐day mortality after STEMI, adjusted for medication use, primary percutaneous coronary intervention (PCI), and other medical conditions, was higher for women than men under 60 years of age.^5^ A large cohort study in the United Kingdom (691 290 people with STEMI) similarly found that women were less likely than men to meet reperfusion time targets or receive antiplatelet therapy, and that adjusted 30‐day mortality was higher for women.^6^

In 2018, we reported that our analysis of CONCORDANCE acute coronary syndrome registry data from 41 Australian hospitals for the period February 2009 – May 2016 indicated that, after adjusting for clinical factors, women with STEMI received less invasive management and less preventive medication at discharge, and that six‐month mortality and major adverse cardiovascular event (MACE) rates were twice as high as for men.^4^

Awareness of sex differences in CVD outcomes has increased over the past two decades, leading to targeted campaigns for improving the recognition of heart disease in women and their outcomes, including the HER Disease Campaign in the United Kingdom,^7^ Go Red for Women (American Heart Association),^8^ and the Women's Hearts: Making the Invisible Visible (Australian Heart Foundation).^9^ These campaigns aim to improve public awareness, change public health policy, improve the education of medical staff, and support sex‐specific heart disease research.

In the context of these new strategies, we examined whether the sex differences in CVD care and outcomes for people hospitalised with STEMI in New South Wales have been reduced during 2011–2020.

## Methods

We undertook a retrospective cohort study, analysing data for all adults (18 years or older) who were admitted to any public or private hospital in New South Wales with STEMI for the first time during 1 January 2011 – 31 December 2020. At the time of the study, New South Wales included about one‐third of the Australian population.^10^ We report our study according to the STROBE statement for reporting cohort studies.^11^

### Data source

Patient data were extracted from the New South Wales Admitted Patient Data Collection (APDC), which comprises patient data from about 400 services, including public and private hospitals, multi‐purpose services, and private day procedure centres.^12^ The dataset, managed by the Centre for Health Record Linkage (CHeReL), includes patient demographic information and hospitals administrative data, including hospital length of stay and diagnosis and procedures data.^13^ Admission diagnoses are coded according to the International Classification of Diseases, tenth revision, Australian modification (ICD‐10‐AM); procedures data are coded according to the Australian Classification of Health Interventions (ACHI).^12^ The patient records were linked to the New South Wales Cause of Death Unit Record File dataset of the Registry of Births, Deaths and Marriages (to 31 December 2020). CheReL performed probabilistic data linkage, with an estimated false positive rate of 0.5%.^13^

We defined STEMI admission as a “continuous period of hospital care represented by linked contiguous hospital records that start with an acute care record and urgency of admission of ‘emergency’ and has either a principal diagnosis of STEMI on the initial record or a cardiac principal diagnosis on the initial record with a subsequent record with a principal diagnosis of STEMI within 24 hours of initial admission”^14^ (ICD‐10‐AM codes: Supporting Information, table 1). This definition took into account any changes to the hospital admission, such as transfer to a PCI facility. We included the first STEMI admission for each person; any subsequent STEMI admissions were excluded.

### Variables and outcomes

The patient socio‐demographic characteristics included in our analyses were age, sex (binary variable, as documented in the dataset), country of birth, and residential postcode‐based socio‐economic position (Index of Relative Socio‐Economic Advantage and Disadvantage [IRSAD]: low scores indicate areas of greatest disadvantage; that is, a large proportion of residents have low incomes or education levels).^15^ As other medical conditions we included cerebrovascular disease, heart failure, diabetes, peripheral vascular disease, dementia, chronic obstructive pulmonary disease, and cancer (extracted using ICD‐10‐AM codes and the R *comorbidity* package^16^). We also calculated the Charlson comorbidity index (CCI) score based on other medical conditions diagnosed during the five years preceding the first STEMI hospitalisation, according to admission records. CCI scores predict ten‐year mortality risk for a hospitalised person (mild, 1 or 2; moderate, 3 or 4; severe, 5 or more).^17^

We calculated the proportions of people who underwent angiography or revascularisation (PCI or coronary artery bypass grafting [CABG]) within seven days of admission, as defined by ACHI codes (Supporting Information, table 1). We assessed MACE and mortality (overall and CVD‐related) within twelve months of discharge from the initial STEMI admission; MACE was defined as cardiovascular death, repeat myocardial infarction, or stroke, based on ICD‐10‐AM codes (Supporting Information, table 2).

### Statistical analysis

The first STEMI admission was identified for each person, and presentations were grouped by calendar year. We summarise patient characteristics by two‐ or three‐year blocks as means with standard deviations (SDs) or numbers and proportions. We assessed the statistical significance of differences between male and female patients using χ^2^ and Wilcoxon rank sum tests, as appropriate. We assessed changes with time in invasive angiography, PCI, MACE, and death rates by sex in binomial regression models with an identity link and adjusted for age group, intensive care unit admission, and CCI score. We report changes in proportion per year with 95% confidence intervals (CIs), and depict the changes in graphs. To assess whether changes differed by sex or age groups (under 75 years, 75 years or older), an interaction term was included in the model; further analyses compared outcomes for patients under 85 years or aged 85 years or older. *P* < 0.05 was deemed statistically significant. Data analysis was performed in R 4.2.3 (R Foundation for Statistical Computing) and the packages *gtsummary*,^18^ *ggplot2*,^19^ and *comorbidity*.^16^

### Ethics approval

The New South Wales Population and Health Services Research Ethics Committee approved the study and waived the requirement for individual consent by patients for the use of their data (2020/ETH03081/2021.38).

## Results

We identified 29 435 initial hospital admissions of people with STEMI during 2011–2020; the mean age at admission was 66.1 years (SD, 14.2 years), 8475 were female patients (28.8%) and 20 960 male patients (71.2%), and 20 256 were born in Australia or New Zealand (68.8%). Age and sex distributions were similar across the study period. A total of 14 296 people (48.6%) lived in areas in the two IRSAD quintiles of greatest disadvantage. At the time of their initial STEMI admissions, 4960 people (16.9%) had histories of diabetes, 4271 (14.5%) of heart failure, 4218 (14.3%) of peripheral vascular disease, and 1492 (5.1%) of cerebrovascular disease. The proportions of people with histories of heart failure, cerebrovascular disease, dementia, chronic pulmonary disease, and diabetes were each higher for female than male patients across the study period. The mean CCI score was 1.9 (SD, 1.3); the mean CCI score was higher for female than for male patients across the study period (Box 1).

The mean age at admission was higher for female than male patients (72.4 [SD, 14.5] *v* 63.6 [SD, 13.3] years), and a larger proportion were aged 85 years or older (2173, 25.6% *v* 1514, 7.2%) (Supporting Information, table 3). A larger proportion of female than of male patients lived in areas in the two IRSAD quintiles of greatest disadvantage (4336, 51.2% *v* 9960, 47.6%). The proportions of people with histories of heart failure, cerebrovascular disease, dementia, chronic pulmonary disease, and diabetes were each higher for female than male patients, and their mean CCI score was higher (2.1 [SD, 1.4] *v* 1.8 [SD, 1.2]) (Box 1).

Box 1 **Demographic characteristics of 29 435 people admitted to New South Wales public or private hospitals with ST‐elevation myocardial infarction (STEMI) for the first time, 1 January 2011 – 31 December 2020, by time period and sex**

**Table:** 

	Admission year				
Characteristic	2011–2013	2014–2016	2017–2018	2019–2020	Total
**Overall**	9072	9280	5663	5420	29 435
**Sex**					
Female	2728 (30.1%)	2657 (28.6%)	1651 (29.2%)	1439 (26.5%)	8475 (28.8%)
Male	6344 (69.9%)	6623 (71.4%)	4012 (70.8%)	3981 (73.5%)	20 960 (71.2%)
Age (years), mean (SD)*	65.9 (14.5)	66.2 (14.3)	66.4 (13.9)	66.1 (13.9)	66.1 (14.2)
Female	72.4 (14.7)	72.7 (14.5)	71.9 (14.1)	72.2 (14.6)	72.4 (14.5)
Male	63.1 (13.4)	63.5 (13.3)	64.1 (13.2)	63.9 (13.0)	63.6 (13.3)
**Country of birth** ^**†**^					
Australia/New Zealand	6302 (71.9%)	6413 (70.9%)	3874 (69.3%)	3666 (68.6%)	20 255 (68.8%)
Female	1996 (75.7%)	1952 (75.1%)	1204 (73.8%)	1046 (73.7%)	6198 (74.8%)
Male	4306 (70.3%)	4461 (69.2%)	2670 (67.4%)	2620 (66.7%)	14 057 (68.7%)
Europe	1395 (15.9%)	1416 (15.7%)	819 (14.6%)	764 (14.3%)	4394 (15.3%)
Female	425 (16.1%)	371 (14.3%)	241 (14.8%)	205 (14.4%)	1242 (15.0%)
Male	970 (15.8%)	1045 (16.2%)	578 (14.6%)	559 (14.2%)	3152 (15.4%)
Asia	551 (6.3%)	673 (7.4%)	511 (9.1%)	509 (9.5%)	2244 (7.8%)
Female	120 (4.6%)	164 (6.3%)	106 (6.5%)	78 (5.5%)	468 (5.6%)
Male	431 (7.0%)	509 (7.9%)	405 (10.2%)	431 (11.0%)	1776 (8.7%)
Other countries	512 (5.8%)	545 (6.0%)	387 (6.9%)	406 (7.6%)	1850 (6.4%)
Female	95 (3.6%)	112 (4.3%)	81 (5.0%)	90 (6.3%)	378 (4.6%)
Male	417 (6.8%)	433 (6.7%)	306 (7.7%)	316 (8.0%)	1472 (7.2%)
Missing data	312	233	72	75	692
Female	92	58	19	20	189
Male	220	175	53	55	503
**Socio‐economic position (IRSAD)** ^**†**^					
Quintile 1 (most disadvantage)	2126 (23.5%)	2175 (23.5%)	1304 (23.0%)	1238 (22.9%)	6841 (23.3%)
Female	688 (25.2%)	656 (24.7%)	397 (24.0%)	349 (24.3%)	2090 (24.7%)
Male	1436 (22.7%)	1519 (23.0%)	907 (22.6%)	889 (22.4%)	4751 (22.7%)
Quintile 2	2426 (26.8%)	2376 (25.6%)	1340 (23.7%)	1313 (24.3%)	7455 (25.4%)
Female	749 (27.5%)	711 (26.8%)	416 (25.2%)	370 (25.7%)	2246 (26.5%)
Male	1677 (26.5%)	1665 (25.2%)	924 (23.1%)	943 (23.7%)	5209 (24.9%)
Quintile 3	1499 (16.5%)	1511 (16.3%)	1024 (18.1%)	968 (17.9%)	5002 (17.0%)
Female	449 (16.5%)	429 (16.1%)	274 (16.6%)	246 (17.1%)	1398 (16.5%)
Male	1050 (16.6%)	1082 (16.4%)	750 (18.7%)	722 (18.2%)	3604 (17.2%)
Quintile 4	1354 (14.9%)	1453 (15.7%)	863 (15.3%)	865 (16.0%)	4537 (15.4%)
Female	384 (14.1%)	393 (14.8%)	261 (15.8%)	222 (15.4%)	1260 (14.9%)
Male	972 (15.3%)	1060 (16.0%)	602 (15.0%)	643 (16.2%)	3277 (15.7%)
Quintile (least disadvantage)	1659 (18.3%)	1756 (18.9%)	1127 (19.9%)	1030 (19.0%)	5572 (18.9%)
Female	458 (16.8%)	468 (17.6%)	303 (18.4%)	251 (17.5%)	1480 (17.5%)
Male	1201 (19.0%)	1288 (19.5%)	824 (20.6%)	779 (19.6%)	4092 (19.5%)
Missing data	8	9	5	6	28
Female	0	0	0	1	1
Male	8	9	5	5	27
**Other medical conditions**					
Heart failure*	1201 (13.2%)	1330 (14.3%)	909 (16.1%)	831 (15.3%)	4271 (14.5%)
Female	546 (20.0%)	554 (20.9%)	370 (22.4%)	301 (20.9%)	1771 (20.9%)
Male	655 (10.3%)	776 (11.7%)	539 (13.4%)	530 (13.3%)	2500 (11.9%)
Peripheral vascular disease*	1340 (14.8%)	1383 (14.9%)	761 (13.4%)	734 (13.5%)	4218 (14.3%)
Female	376 (13.8%)	339 (12.8%)	215 (13.0%)	180 (12.5%)	1110 (13.1%)
Male	964 (15.2%)	1044 (15.8%)	546 (13.6%)	554 (13.9%)	3108 (14.8%)
Cerebrovascular disease*	313 (3.5%)	496 (5.3%)	339 (6.0%)	344 (6.3%)	1492 (5.1%)
Female	133 (4.9%)	211 (7.9%)	139 (8.4%)	124 (8.6%)	607 (7.2%)
Male	180 (2.8%)	285 (4.3%)	200 (5.0%)	220 (5.5%)	885 (4.2%)
Dementia*	99 (1.1%)	220 (2.4%)	185 (3.3%)	202 (3.7%)	706 (2.4%)
Female	50 (1.8%)	112 (4.2%)	100 (6.1%)	105 (7.3%)	367 (4.3%)
Male	49 (0.8%)	108 (1.6%)	85 (2.1%)	97 (2.4%)	339 (1.6%)
Chronic pulmonary disease*	366 (4.0%)	660 (7.1%)	609 (10.8%)	608 (11.2%)	2243 (7.6%)
Female	165 (6.0%)	263 (9.9%)	243 (14.7%)	215 (14.9%)	886 (10.5%)
Male	201 (3.2%)	397 (6.0%)	366 (9.1%)	393 (9.9%)	1357 (6.5%)
Diabetes*	823 (9.1%)	1755 (18.9%)	1193 (21.1%)	1189 (21.9%)	4960 (16.9%)
Female	260 (9.5%)	535 (20.1%)	365 (22.1%)	364 (25.3%)	1524 (18.0%)
Male	563 (8.9%)	1220 (18.4%)	828 (20.6%)	825 (20.7%)	3436 (16.4%)
Cancer	271 (3.0%)	515 (5.5%)	406 (7.2%)	461 (8.5%)	1653 (5.6%)
Female	73 (2.7%)	153 (5.8%)	122 (7.4%)	127 (8.8%)	475 (5.6%)
Male	198 (3.1%)	362 (5.5%)	284 (7.1%)	334 (8.4%)	1178 (5.6%)
Charlson comorbidity index score, mean (SD)*	1.6 (1.0)	1.9 (1.3)	2.1 (1.4)	2.1 (1.4)	1.9 (1.3)
Female	1.8 (1.1)	2.1 (1.4)	2.4 (1.5)	2.4 (1.6)	2.1 (1.4)
Male	1.6 (1.0)	1.8 (1.2)	2.0 (1.3)	2.0 (1.4)	1.8 (1.2)

IRSAD = Index of Relative Socio‐Economic Advantage and Disadvantage; SD = standard deviation.

* Female *v* male patients: *P* < 0.001.

† For overall category: *P* < 0.001.

### Procedures

A total of 23 939 people (81.3%) underwent coronary angiography, 19 294 (65.5%) underwent PCI, and 1606 (5.5%) underwent CABG within seven days of index STEMI admissions. The proportion of female patients who underwent angiography was smaller than that of male patients (71.9% *v* 85.1%), as were the proportions who underwent PCI (54.4% *v* 70.0%) or CABG (3.3% *v* 6.3%) (Box 2).

The proportion of patients who underwent angiography within seven days of first STEMI admission increased from 75.5% in 2011–2013 to 89.7% in 2019–2020; the proportion who underwent PCI increased from 46.4% to 67.4% (Box 2). The increases were more rapid for female than male patients (Box 3). After adjusting for age, intensive care unit admission, and CCI score, the angiography proportion increased by 2.7 (95% CI, 2.5–2.9) percentage points per year for female patients, and 1.5 (95% CI, 1.4–1.6) percentage points per year for male patients; the PCI proportion increased by 3.2 (95% CI, 2.9–3.6) percentage points per year for female patients and 2.5 (95% CI, 2.3–2.7) percentage points per year for male patients (Box 4).

Box 2 **Procedures and outcomes for 29 435 people admitted to New South Wales public or private hospitals with ST‐elevation myocardial infarction (STEMI) for the first time, 1 January 2011 – 31 December 2020, by time period and sex**

**Table:** 

	Admission year				
Characteristic	2011–2013	2014–2016	2017–2018	2019–2020	Total
**Total number of patients**	9072	9280	5663	5420	29 435
**Sex**					
Female	2728 (30.1%)	2657 (28.6%)	1651 (29.2%)	1439 (26.5%)	8475 (28.8%)
Male	6344 (69.9%)	6623 (71.4%)	4012 (70.8%)	3981 (73.5%)	20 960 (71.2%)
**Procedures (within seven days of admission)**					
Angiography*	6852 (75.5%)	7374 (79.5%)	4852 (85.7%)	4861 (89.7%)	23 939 (81.3%)
Female	1794 (65.8%)	1836 (69.1%)	1289 (78.1%)	1173 (81.5%)	6092 (71.9%)
Male	5058 (79.7%)	5538 (83.6%)	3563 (88.8%)	3688 (92.6%)	17 847 (85.1%)
Percutaneous coronary intervention*	5254 (57.9%)	5890 (63.5%)	3988 (70.4%)	4162 (76.8%)	19 294 (65.5%)
Female	1266 (46.4%)	1372 (51.6%)	1005 (60.9%)	970 (67.4%)	4613 (54.4%)
Male	3988 (62.9%)	4518 (68.2%)	2983 (74.4%)	3192 (80.2%)	14 681 (70.0%)
Coronary artery bypass grafting*	511 (5.6%)	535 (5.8%)	301 (5.3%)	259 (4.8%)	1606 (5.5%)
Female	103 (3.8%)	84 (3.2%)	47 (2.8%)	44 (3.1%)	278 (3.3%)
Male	408 (6.4%)	451 (6.8%)	254 (6.3%)	215 (5.4%)	1328 (6.3%)
**Outcomes (within twelve months of discharge)**					
Admitted to intensive care (during admission)*	728 (8.0%)	733 (7.9%)	576 (10.2%)	645 (11.9%)	2682 (9.1%)
Female	200 (7.3%)	203 (7.6%)	146 (8.8%)	139 (9.7%)	688 (8.1%)
Male	528 (8.3%)	530 (8.0%)	430 (10.7%)	506 (12.7%)	1994 (9.5%)
Major adverse cardiovascular event*	1592 (17.5%)	1498 (16.1%)	885 (15.6%)	720 (13.3%)	4695 (16.0%)
Female	564 (20.7%)	491 (18.5%)	276 (16.7%)	226 (15.7%)	1557 (18.4%)
Male	1028 (16.2%)	1007 (15.2%)	609 (15.2%)	494 (12.4%)	3138 (15.0%)
Cardiovascular death*	623 (6.9%)	469 (5.1%)	167 (2.9%)	85 (1.6%)	1344 (4.6%)
Female	286 (10.5%)	197 (7.4%)	72 (4.4%)	40 (2.8%)	595 (7.0%)
Male	337 (5.3%)	272 (4.1%)	95 (2.4%)	45 (1.1%)	749 (3.6%)
All‐cause mortality*	1436 (15.8%)	1035 (11.2%)	366 (6.5%)	188 (3.5%)	3025 (10.3%)
Female	602 (22.1%)	420 (15.8%)	139 (8.4%)	81 (5.6%)	1242 (14.7%)
Male	834 (13.1%)	615 (9.3%)	227 (5.7%)	107 (2.7%)	1783 (8.5%)

* Female *v* male patients (2011–2020): *P* < 0.001.

Box 3 **Proportions of people who underwent coronary angiography or percutaneous coronary interventions within seven days of first hospital admission with ST‐elevation myocardial infarction (STEMI), New South Wales, 1 January 2011 – 31 December 2020, by sex**

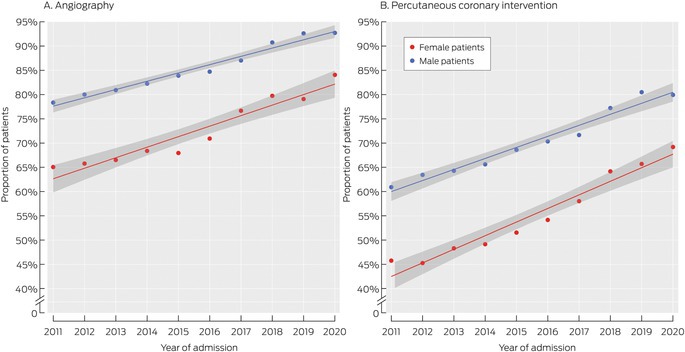

The rates of change in angiography and PCI proportions were also greater for female than male patients when assessed separately for people under 85 years of age or aged 85 years or older; differences between male and female patients were closing more rapidly for those under 85 years of age than for those aged 85 years or older (Supporting Information, figures 1 and 2).

### Outcomes

The proportion of female patients who were admitted to intensive care units was smaller than for male patients (8.1% *v* 9.5%). The proportions of first STEMI admissions followed by MACE (18.4% *v* 15.0%), cardiovascular death (7.0% *v* 3.6%), or death from any cause (14.7% *v* 8.5%) were each larger for female than male patients (Box 2).

Within twelve months of discharge from first STEMI admissions, 4695 people (16.0%) had experienced MACE and 3025 (10.3%) had died (any cause). The proportion of patients who experienced MACE declined from 15.5% in 2011–2013 to 13.3% in 2019–2020; the proportion who died (any cause) declined from 15.8% to 3.5% (Box 2). The declines in mortality proportion were more rapid for female than male patients; those for MACE were similar (Box 5). After adjusting for age, intensive care unit admission, and CCI score, the MACE proportion declined by 0.8 (95% CI, 0.5–1.1) percentage points per year for female patients and 0.5 (95% CI, 0.3–0.7) percentage points per year for male patients; the cardiovascular death rate declined by 0.4 (95% CI, 0.3–0.4) percentage points per year for female patients and 0.2 (95% CI, 0.2–0.2) percentage points per year for male patients, and the all‐cause mortality proportion declined by 1.0 (95% CI, 0.8–1.1) percentage points per year for female patients and 0.6 (95% CI, 0.5–0.7) percentage points per year for male patients (Box 4).

Box 4 **Changes in proportions of procedures and outcomes for people admitted to New South Wales public or private hospitals with ST‐elevation myocardial infarction (STEMI) for the first time, 1 January 2011 – 31 December 2020, by sex: adjusted binomial regression analysis**

**Table:** 

Parameter	Change per year, percentage points (95% confidence interval)		
	Female patients	Male patients	*P* (interaction)
Procedure (within seven days of admission)			
Angiography	2.69 (2.47 to 2.90)	1.49 (1.35 to 1.63)	< 0.001
Percutaneous coronary intervention	3.22 (2.87 to 3.57)	2.54 (2.34 to 2.74)	0.001
Outcome (within twelve months of discharge)			
Major adverse cardiovascular event	–0.77 (–1.05 to –0.50)	–0.50 (–0.66 to –0.33)	0.084
Death (cardiovascular disease)	–0.37 (–0.45 to –0.28)	–0.21 (–0.25 to –0.17)	0.001
Death (all causes)	–0.98 (–1.11 to –0.85)	–0.59 (–0.66 to 0.52)	< 0.001

Box 5 **Major adverse cardiovascular events (MACE) and all‐cause mortality within twelve months of discharge from first hospital admission with ST‐elevation myocardial infarction (STEMI), New South Wales, 1 January 2011 – 31 December 2020, by sex**

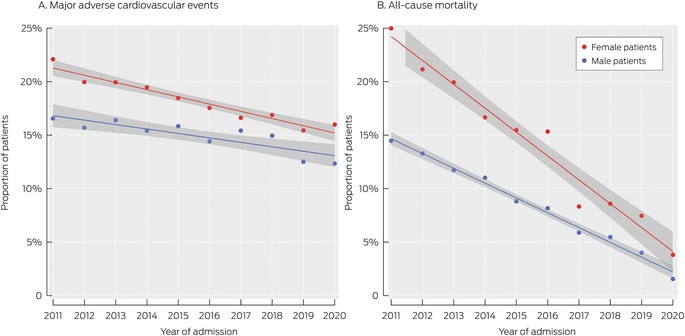

The sex difference in the decline in MACE proportion was not statistically significant for people under 85 years of age or for those aged 85 years or older; the rates of decline of CVD and all‐cause mortality were significantly greater for female patients than male patients under 85 years of age, but not for those aged 85 years or older (Supporting Information, table 4).

## Discussion

We reported in 2018 that female patients hospitalised with STEMI were less likely than male patients to receive invasive management and more likely to die within six months of admission.^4^ Our new analysis broadly confirms our earlier findings, but we also found that these differences declined during 2011–2020. We found that increases in the proportions of people undergoing timely angiography or PCI were greater for female than male patients, and that the declines in the proportions of admissions followed by MACE, cardiovascular death, or death of any cause were greater for female patients. We report the first evidence that sex differences in treatments and outcomes for people with STEMI can be reduced.

Research during the past decade has consistently found sex differences in the management of acute coronary syndrome; women are less likely to undergo angiography or PCI and have poorer outcomes than men.^4^ A large United States study (177 602 female patients) found that the number of STEMI hospitalisations of women under 45 years of age increased during 2008–2019; in‐hospital mortality for women under 55 years of age with STEMI had not changed, despite the overall national decline in in‐hospital mortality associated with STEMI admissions (from 12.4% to 10.5%).^20^ A large meta‐analysis of studies from thirty countries (707 098 patients, 31% women) found that a smaller proportion of female than male patients received primary PCI (59.5% *v* 68.2%),^21^ despite the recognised acute coronary syndrome management benefits for both sexes.

Lower diagnosis and treatment rates and poorer outcomes for women with CVD have been reported for 25 years. There is growing appreciation and knowledge of sex differences in the clinical presentation, risk factors, and aetiology of CVD that influence the likelihood of CVD in women being recognised and managed efficiently.^22^ Sex‐specific symptoms and risk factors, such as a history of gestational diabetes, pre‐eclampsia, and early menopause, can be overlooked when assessing women for coronary artery disease.^23^ Conditions such as myocardial infarction with non‐obstructive coronary arteries (MINOCA) and spontaneous coronary artery dissection are more likely as causes of acute coronary syndrome in women.^24^ Further, the mean age and number of other medical conditions at presentation with acute coronary syndrome are both higher for women than men, which may partly explain differences in their outcomes.^25^, ^26^

It is difficult to evaluate the impact of changing knowledge, but increasing awareness of sex differences in treatment and outcomes could influence the clinical practice of doctors investigating patients for CVD, and this could explain the findings of our analysis. Many large national cardiovascular organisations conduct campaigns for raising awareness of heart disease in female patients among both the public and clinicians, and provide information about how to improve its assessment and management.

Despite sex differences in CVD characteristics, specific measures can improve outcomes for all patients. A study at a Cleveland clinic (1272 participants; 2014–2016) found that a four‐step protocol with a systems‐based approach to STEMI reduced sex differences and improved outcomes for women: activation of the emergency department catheterisation laboratory, a STEMI safe handoff checklist, prompt transfer to an immediately available catheterisation laboratory, and the radial‐first approach to PCI. The investigators assessed guideline‐directed medical therapy, door‐to‐balloon time, in‐hospital adverse events, and 30‐day mortality before and after the introduction of their protocol. The difference between women and men in all‐cause mortality was reduced from 6.1% higher to 3.2% higher in women after the protocol was implemented, and improvements in guideline‐directed medical therapy and door‐to‐balloon time were also reported.^27^

Other measures, such as a network system for primary PCI, could also improve outcomes for all patients. The introduction of a primary PCI protocol and increased access to primary PCI centres across New South Wales may have contributed to the improvements in reperfusion, MACE, and mortality we found. A Spanish study similar to ours that examined the impact of the introduction of a network system for primary PCI study (277 281 patients with STEMI) found that the proportion of patients who received primary PCI increased during 2005–2015 from 34.9% to 68.1% in men and from 21.7% to 51.7% in women; however, major differences for reperfusion therapy (men *v* women, 2005: 56.6% *v* 36.4%; 2015: 75.6% *v* 57.0%) remained.^28^

Other strategies for reducing sex differences in outcomes for women with CVD include specialised cardiology clinics for women, further research into cardiovascular disease in female patients, and wider recognition of sex differences in medical education and at medical conferences. Sex‐specific differences have been incorporated into European Society of Cardiology guidelines, to “highlight the fact that women and men receive equal benefit from a reperfusion strategy and STEMI‐related therapy, and that both genders must be managed in a similar fashion.”^29^ The American College of Cardiology/American Heart Association has also emphasised the importance of acknowledging sex differences.^25^ Our findings suggest that strategies for overcoming sex differences in the management of acute coronary syndrome are succeeding.

### Limitations

Our analysis of linked administrative data was based on hospital coding; detailed clinical information was therefore not available. Changes to coding during 2011–2020 are possible, but the only change known to us is that the reporting of diabetes became mandatory in 2012, explaining the larger proportions of patients with diabetes in our study after 2011–2013. People who lived near the New South Wales border could have been referred to interstate hospitals for primary PCI, data for which would not be included in our dataset. The problem of potentially missing data from patients referred interstate was discussed by the authors of another study based on the New South Wales STEMI dataset, who compared New South Wales, interstate, and national data.^14^ We believe that these study limitations would not have affected our research findings, as they would equally affect data for both sexes.

### Conclusion

Given the magnitude of the sex‐related differences in the treatment and outcomes for people with STEMI, the rates of improvement we found will not be sufficient to completely close them during the next ten years. They do, however, indicate that they can be narrowed and that we have the tools to do so. Further investigation of the strategies that have led to these improvements is needed to further improve outcomes for female patients with STEMI.

## Open access

Open access publishing facilitated by The University of Sydney, as part of the Wiley – The University of Sydney agreement via the Council of Australian University Librarians.

## Competing interests

No relevant disclosures.

## Data sharing

The de‐identified data we analysed are not publicly available, but requests to the corresponding author for the data will be considered on a case‐by‐case basis.

## Author contributions

Clara Chow provided resources, was responsible for the research design, data analysis, validation, methodology, formal analysis, and supervised the project and manuscript composition. Samia Kazi obtained data access and ethics approval, was responsible for research design, methodology, data analysis, and validation, and drafted the initial manuscript. Simone Marschner, Haeri Min, and Desi Quintans contributed to methodology, software, data analysis, and validation, and contributed to manuscript development. James Chong, Ehsan Khan, and David Brieger contributed to the final manuscript. All authors agreed on the final manuscript before submission.

Received 21 January 2025, accepted 10 June 2025

## Supporting information


Supplementary methods and results
