# Supplementary material for: Sex differences in management and outcomes of people with ST‐elevation myocardial infarction, New South Wales, 2011–2020: a retrospective cohort study
Source: Med J Aust. 2025 Sep 18;223(7):351–8. doi: 10.5694/mja2.70048 (PMC12502883; doi:10.5694/mja2.70048)
Supplement: Supplementary file 1 — Supplementary methods and results [file MJA2-223-351-s001.pdf]

The Medical Journal of Australia

Supporting Information

Supplementary methods and results

This appendix was part of the submitted manuscript and has been peer reviewed.
It is posted as supplied by the authors.

Appendix to: Kazi S, Marschner S, Min H, et al. Sex differences in management and outcomes of people with ST-elevation myocardial infarction, New South Wales, 2011–2020: a retrospective cohort study. *Med J Aust* 2025; doi: 10.5694/mja2.70048.

Supplementary methods

Table 1. International Classification of Diseases, tenth revision, Australian modification (ICD-10-AM) and Australian Classification of Health Interventions (ACHI) codes used in the New South Wales Admitted Patient Data Collection

ST-elevation myocardial infarction (STEMI) principal diagnosis: I21.0 – I21.3, I22.0, I22.1, I22.8<br>OR<br>Cardiac principal diagnosis (I00-I02, I05-09, I10-I15, I20-I25, I30- I52, I70-I72, I74, I77.0, R00, R01, R03, R07) with AND within 24 hours of admission has a subsequent principal diagnosis of STEMI (I21.0 – I21.3, I22.0, I22.1, I22.8)
Angiography: procedure codes within 7 days of STEMI:<br>(ACHI codes: 38300-00, 38303-00, 38306-00, 38306-01, 38306-02, 38309-00, 38312-00, 38312-01, 38315-00, 38318-00, 38318-01, 90218-00, 90218-01, 90218-02, 90218-03, 38215-00, 38218-00, 38218-01, 38218-02)
PCI: procedure codes within 7 days of STEMI<br>(ACHI codes: 38300-00, 38303-00, 38306-00, 38306-01, 38306-02, 38309-00, 38312-00, 38312-01, 38315-00, 38318-00, 38318-01, 90218-00, 90218-01, 90218-02, 90218-03)
Cardiovascular death: I10 codes from the underlying cause of death variable
MACE: STEMI codes or death code (i.e. I10 codes from the underlying cause of death variable in the mortality data) or MACE ICD-10-AM (see supplementary table 2)

MACE: major adverse cardiovascular event; PCI: percutaneous coronary intervention; STEMI: ST-elevation myocardial infarction.

Table 2. International Classification of Diseases, tenth revision, Australian modification (ICD-10-AM) codes used in the New South Wales Admitted Patient Data Collection to define major adverse cardiovascular events (MACE)

ICD code description for major adverse cardiovascular events:
I11.0 Hypertensive heart disease with (congestive) heart failure
I13.0 Hypertensive heart and kidney disease with (congestive) heart failure
I13.2 Hypertensive heart and kidney disease with both (congestive) heart failure and kidney failure
I21 Acute myocardial infarction
I21.0 Acute transmural myocardial infarction of anterior wall
I21.1 Acute transmural myocardial infarction of inferior wall
I21.2 Acute transmural myocardial infarction of other sites
I21.3 Acute transmural myocardial infarction of unspecified site
I21.4 Acute subendocardial myocardial infarction
I21.9 Acute myocardial infarction, unspecified
I46 Cardiac arrest
I46.0 Cardiac arrest with successful resuscitation
I46.1 Sudden cardiac death, so described
I46.9 Cardiac arrest, unspecified
I50 Heart failure
I50.0 Congestive heart failure
I50.1 Left ventricular failure
I50.9 Heart failure, unspecified
I61 Intracerebral haemorrhage
I61.0 Intracerebral haemorrhage in hemisphere, subcortical
I61.1 Intracerebral haemorrhage in hemisphere, cortical
I61.2 Intracerebral haemorrhage in hemisphere, unspecified
I61.3 Intracerebral haemorrhage in brain stem
I61.4 Intracerebral haemorrhage in cerebellum
I61.5 Intracerebral haemorrhage, intraventricular
I61.6 Intracerebral haemorrhage, multiple localised
I61.8 Other intracerebral haemorrhage
I61.9 Intracerebral haemorrhage, unspecified
I63 Cerebral infarction
I63.0 Cerebral infarction due to thrombosis of precerebral arteries
I63.1 Cerebral infarction due to embolism of precerebral arteries
I63.2 Cerebral infarction due to unspecified occlusion or stenosis of precerebral arteries
I63.3 Cerebral infarction due to thrombosis of cerebral arteries
I63.4 Cerebral infarction due to embolism of cerebral arteries
I63.5 Cerebral infarction due to unspecified occlusion or stenosis of cerebral arteries
I63.6 Cerebral infarction due to cerebral venous thrombosis, non-pyogenic
I63.8 Other cerebral infarction
I63.9 Cerebral infarction, unspecified
I64 Stroke, not specified as haemorrhage or infarction

Supplementary results

Table 3. Demographic characteristics of 29 435 people admitted to New South Wales public or private hospitals with ST-elevation myocardial infarction (STEMI) for the first time, 1 January 2011 – 31 December 2020, by sex

Characteristic	Female patients	Male patients	Total
Total number of patients*	8475 (28.8%)	20 960 (71.2%)	29 435
Age (years), mean (SD)*	72.4 (14.5)	63.6 (13.3)	66.1 (14.2)
Age group (years)†			
Under 35	41 (0.5%)	198 (0.9%)	239 (0.8%)
35–44	199 (2.3%)	1191 (5.7%)	1390 (4.7%)
45–54	829 (9.8%)	3774 (18.0%)	4603 (15.6%)
55–64	1404 (16.6%)	5789 (27.6%)	7193 (24.4%)
65–74	1804 (21.3%)	5301 (25.3%)	7105 (24.1%)
75–84	2025 (23.9%)	3193 (15.2%)	5218 (17.7%)
85 or older	2173 (25.6%)	1514 (7.2%)	3687 (12.5%)
Country of birth†			
Australia/New Zealand	6198 (74.8%)	14 057 (68.7%)	20 255 (70.5%)
Europe	1242 (15.0%)	3152 (15.4%)	4394 (15.3%)
Asia	468 (5.6%)	1776 (8.7%)	2244 (7.8%)
Other countries	378 (4.6%)	1472 (7.2%)	1850 (6.4%)
Missing data	189	503	692
Socio-economic position (IRSAD)†			
Quintile 1 (most disadvantage)	2090 (24.7%)	4751 (22.7%)	6841 (23.3%)
Quintile 2	2246 (26.5%)	5209 (24.9%)	7455 (25.4%)
Quintile 3	1398 (16.5%)	3604 (17.2%)	5002 (17.0%)
Quintile 4	1260 (14.9%)	3277 (15.7%)	4537 (15.4%)
Quintile 5 (least disadvantage)	1480 (17.5%)	4092 (19.5%)	5572 (18.9%)
Missing data	1	27	28
Other medical conditions			
Heart failure*	1771 (20.9%)	2500 (11.9%)	4271 (14.5%)
Peripheral vascular disease*	1110 (13.1%)	3108 (14.8%)	4218 (14.3%)
Cerebrovascular disease*	607 (7.2%)	885 (4.2%)	1492 (5.1%)
Dementia*	367 (4.3%)	339 (1.6%)	706 (2.4%)
Chronic pulmonary disease*	886 (10.5%)	1357 (6.5%)	2243 (7.6%)
Diabetes*	1524 (18.0%)	3436 (16.4%)	4960 (16.9%)
Cancer	475 (5.6%)	1178 (5.6%)	1653 (5.6%)
Charlson comorbidity index score, mean (SD)*	2.1 (1.4)	1.8 (1.2)	1.9 (1.3)
Procedures (within seven days of admission)			
Angiography*	6092 (71.9%)	17 847 (85.1%)	23 939 (81.3%)
Percutaneous coronary intervention*	4613 (54.4%)	14 681 (70.0%)	19 294 (65.5%)
Coronary artery bypass grafting*	278 (3.3%)	1328 (6.3%)	1606 (5.5%)
Outcomes (within twelve months of discharge)			
Admitted to intensive care (during admission)*	688 (8.1%)	1994 (9.5%)	2682 (9.1%)
Major adverse cardiovascular event*	1557 (18.4%)	3138 (15.0%)	4695 (16.0%)
Cardiovascular death*	595 (7.0%)	749 (3.6%)	1344 (4.6%)
All-cause mortality*	1242 (14.7%)	1783 (8.5%)	3025 (10.3%)

IRSAD = Index of Relative Socio-Economic Advantage and Disadvantage; SD = standard deviation.

* Female *v* male patients: $P < 0.001$.

† For overall category: $P < 0.001$.

Figure 1. Angiography proportions (with 95% confidence region), by sex and age group

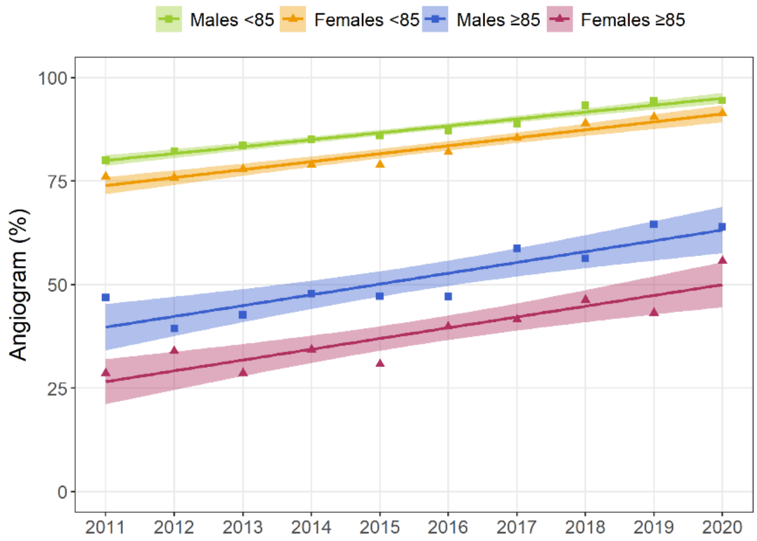

	2011	2012	2013	2014	2015	2016	2017	2018	2019	2020
Males <85	80.1	82.2	83.7	85.0	86.0	87.2	88.9	93.3	94.3	94.5
Females <85	76.1	75.8	77.9	78.9	78.9	82.1	85.4	88.9	90.5	91.4
Males ≥85	46.9	39.4	42.7	47.8	47.2	47.1	58.8	56.3	64.5	63.9
Females ≥85	28.6	33.9	28.6	34.3	30.8	39.9	41.6	46.3	43.2	55.8

Figure 2. Percutaneous coronary intervention proportions (with 95% confidence region), by sex and age group

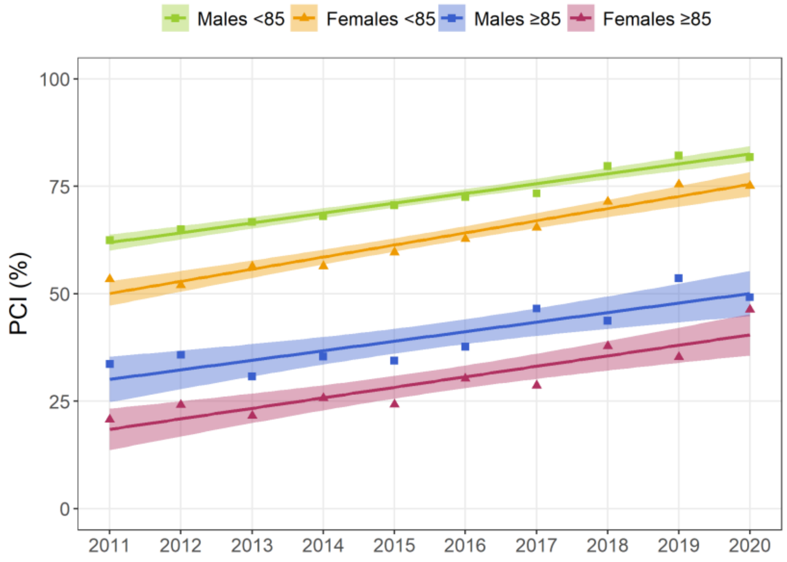

	2011	2012	2013	2014	2015	2016	2017	2018	2019	2020
Males <85	62.4	65.0	66.7	68.1	70.6	72.5	73.4	79.7	82.1	81.8
Females <85	53.4	52.0	56.3	56.4	59.6	62.7	65.4	71.4	75.5	75.1
Males ≥85	33.6	35.8	30.8	35.4	34.4	37.7	46.6	43.7	53.6	49.2
Females ≥85	20.7	24.1	21.6	25.7	24.2	30.3	28.7	37.8	35.2	46.3

Table 3. Changes in proportions of procedures and outcomes for people admitted to New South Wales public or private hospitals with ST elevation myocardial infarction (STEMI) for the first time, 1 January 2011 – 31 December 2020, by sex: adjusted binomial regression analysis; by age group

Parameter	Male patients (95% CI)	Female patients (95% CI)	*P*
**Under 85 years of age**			
Procedure			
PCI within 7 days	2.35% (1.50% to 3.21%)	2.83% (2.43% to 3.23%)	0.038
Angiogram within 7 days	1.66% (1.01% to 2.31%)	1.96% (1.65% to 2.27%)	0.09
Outcome			
MACE within 12 months	–0.36% (–1.03% to 0.31%)	–0.41% (–0.72% to –0.09%)	0.78
CVD death within 12 months	–0.47% (–0.77% to –0.17%)	–0.75% (–0.89% to –0.61%)	<0.001
All-cause mortality within 12 months	–1.26% (–1.73% to –0.78%)	–1.76% (–1.99% to –1.53%)	<0.001
**Aged 85 years or older**			
Procedure			
PCI within 7 days	2.19% (0.31% to 4.07%)	2.20% (1.50% to 2.91%)	0.99
Angiogram within 7 days	2.60% (0.63% to 4.56%)	2.42% (1.67% to 3.17%)	0.78
Outcome			
MACE within 12 months	–1.54% (–3.31% to 0.23%)	–1.48% (–2.2% to –0.80%)	0.91
CVD death within 12 months	–2.41% (–3.77% to –1.04%)	–1.98% (–2.51% to –1.44%)	0.31
All-cause mortality within 12 months	–3.86% (–5.53% to –2.19%)	–3.55% (–4.19% to –2.91%)	0.54

CVD: cardiovascular disease; MACE: major adverse cardiovascular event; PCI: percutaneous coronary intervention.
